# Climate change causes changes in biochemical markers of kidney disease

**DOI:** 10.1186/s12882-020-02186-w

**Published:** 2020-12-12

**Authors:** Richard Kobina Dadzie Ephraim, Christopher Amey Asamoah, Albert Abaka-Yawson, Precious Kwablah Kwadzokpui, Samuel Adusei

**Affiliations:** 1 Department of Medical Laboratory Sciences, School of Allied Health Sciences, University of Cape Coast, Cape Coast, Ghana; 2 Department of Medical Laboratory Sciences, School of Allied Health Sciences, University of Health and Allied Sciences, Ho, Ghana; 3 Department of Obstetrics and Gynaecology, University of Cape Town, Cape Town, South Africa

**Keywords:** Climate change, Kidney disease, Biochemical markers

## Abstract

**Background:**

Climate change is a significant threat to the health of the Ghanaian people. Evidence abounds in Ghana that temperatures in all the ecological zones are rising, whereas rainfall levels have been generally reducing and patterns are increasingly becoming erratic. The study estimated the impact of climate variation between seasons on biochemical markers of kidney disease.

**Methods:**

This study conveniently recruited 50 apparently healthy peasant farmers and hawkers at Wa in the Upper West Region of Ghana. A pre-study screening for hepatitis A and C, Diabetes mellitus, hypertension was done. Serum creatinine and urea levels were analyzed to rule out kidney preexisting kidney disease. Baseline data was collected by estimating urea, creatinine, sodium, potassium, eGFR (estimated glomerular filtration rate) as well as for hemoglobin (Hb) and hematocrit (Hct) concentrations. Anthropometric data such as height, weight and blood pressure were measured by trained personnel. The study participants were closely followed and alerted deep in the dry season for the second sampling (urea, creatinine, hemoglobin, hematocrit, blood pressure, anthropometry).

**Results:**

This study recruited more males (58.82%) than females (41.15%), majority (52.92%) of which were aged 25–29 years with the youngest being 22 years and the eldest being 35 years. The study found body mass index (*p* < 0.001), systolic blood pressure (*p* = 0.019), creatinine (*p* < 0.001), urea (*p* = 0.013) and eGFR (*p* < 0.001) to be significantly influenced by climate change. Stage 1 hypertension was predominant among the study participants during the dry season, 8 (15.69%) than was observed during the rainy season, 4 (7.84%) nonetheless the number of participants with normal BMI rose from 49.02% in the rainy season to 62.75% during the dry reason. Additionally, the study observed that the impact of climate change on systolic blood pressure and urea varied based on age and sex.

**Conclusion:**

This study revealed that climatic changes cause variations in various biochemical parameters used to assess kidney function. Public health education on climatic changes and its implication including precautionary measures should be done among inhabitants of Wa and its environs to reduce its effect. Additionally, appropriate dietary patterns should also be advised to avoid the development of non-communicable diseases such as hypertension and obesity that are known principal causes of Chronic Kidney Disease (CKD).

## Background

Considering the persistent peril of global warming, physicians in recent times have given much attention to the influence of climate change on human health and health care in general [1]. The impacts of human-induced climate alteration are on the upsurge nationwide if not worldwide, threatening human health and the very existence of humanity by affecting our food and water sources, the air we breathe, the weather, and our interactions with the natural environment [2, 3]. Studies suggests that whereas a preponderance of these effects are already being experienced, in the absence of climate change restraint or control, their progression will to a very large extent amplify the existing global health challenges and inequalities so much so that it threatens to undermine principally the social, economic and environmental drivers of health; factors which have contributed largely to human growth and progress [4]. Signals in Ghana indicate that temperatures in all the ecological zones are increasing whilst patterns and levels of rainfall generally are plummeting and increasingly becoming erratic [5]. A study conducted in Adelaide, a city with temperate climate to investigate the association between temperature and admissions for specific kidney diseases thus urolithiasis, urinary tract infections (UTIs) and CKD saw that increases in daily temperature per 1 °C were associated with an increased incidence for all kidney disease categories considered for the study except for pyelonephritis [6]. Similar studies documented an increase in admissions for kidney disease and acute kidney injury during the periods of heat waves compared with non-heat wave periods [7]. Meanwhile according to United Nations Environment Program/United Nations Development Program (UNEP/UNDP), the average annual temperature has over the last 30 years increased 1 °C [8] elevating the risk of kidney pathologies among the Ghanaian people. Worsening the situation however is a predicted average temperature rise of about 0.6 °C, 2.0 °C and 3.9 °C by the year 2020, 2050 and 2080 respectively, in all forest ecological zones coupled with a predicted nose dive of average rainfall of 2.8, 10.9 and 18.6% by 2020, 2050 and 2080 respectively in all forest ecological zones [5]. Richard and colleagues in their work indicated that heat stress doubles the risk for developing CKD among those working in hot environments and that despite the fact that involvement of toxins and infections were not entirely ruled out, common risk factors for each of the CKD epidemics observed in India, Sri Lanka, Mexico, and Central America were hot temperatures and recurrent dehydration that can be linked with climate change [9]. Little data exist on the effect of climate change on kidney function in Ghana hence the necessity for this study.

## Methods

### Study site

This study was conducted at Wa in the Upper West Region of Ghana. The Upper West Region is located in the north-western part of Ghana and shares borders with the La cote D’Ivoire to the north-west, Burkina Faso to the north, Upper East to the East and Northern Region to the South. Wa Municipality with GPS coordinates of 10.0601^o^ N, 2.5099^o^ W has a projected population of 126,609 people as at September, 2018 according to the Ghana Statistical Service (GSS). The climate of the Wa Municipality is characterized by long, windy and hot dry season followed by the short and stormy wet season. The dry season occurs between November and April. The hot seasons records high temperatures with a peak between 40 °C to 45 °C in March and April whereas the wet season records low temperatures with a peak between 21 °C to 23 °C in July to September.

### Study design and eligibility criteria

The study recruited 51 conveniently sampled peasant farmers and hawkers at the market place who were considered “active members of society” based on their occupational activities. A pre-study screening for hepatitis A and C, diabetes mellitus, hypertension was done. Serum creatinine and urea levels were analyzed to rule out kidney disease. Apparently healthy individuals within the ages of 18 to 40 years were recruited for this study. Individuals with hypertension, diabetes mellitus, HIV and kidney diseases individuals were exempted from the study. The study was a longitudinal cohort study where same participants who passed the eligibility criteria were followed up into the dry season and repeat samples taken again. Baseline demographic data (age, gender, weight, height), clinical (systolic and diastolic blood pressure) biochemical (urea, creatinine, urine protein and eGFR), and hematological (Hb and HCT) was collected in the wet season. The study participants were then followed keenly into the dry season and then alerted at the peak of the season for the second data collection.

### Data collection

#### Demographic and anthropometric measurements

Demographic data comprising participant’s age and sex were obtained. Anthropometric data which includes height, weight and hemodynamic data thus blood pressure was determined using specific instrument relating to each parameter. A fully automated blood pressure monitor (Omron Automated Blood Pressure Monitor, HEM-71217, Japan) was used to measure blood pressure (BP) after the participants had sat quietly for at least ten (10) minutes. The BP measurement was taken on the left arm in a seated position, with the arm supported at heart level. Multipurpose weight and height scale was used to measure body weight of the participants to the nearest 0.1 kg and height to the nearest 0.1 cm, with participants standing erect, back straight, heels together, barefooted, and in tight clothing.

#### Blood and urine collection and processing

Eight (8) ml of venous blood sample was collected from the participant’s median cubital vein after overnight fast (8–12 h). Four (4) ml was dispensed into a serum separator tube and 4 ml into EDTA. The blood collected in the serum separator tubes were centrifuges at 3000 rpm for 5 min post clotting. The obtained serum was aliquoted into 2 ml Eppendorf tubes and stored at − 20 °C prior to analysis. The EDTA containing blood was used to a run for full blood count (FBC).

#### Biochemical analysis of blood samples

The serum was analyzed for creatinine and urea. Serum creatinine was determined using the Jaffe reaction method [10], electrolytes were measured by direct electrochemical method using ion selective electrodes and urea by enzymatic – UV – kinetic method using (BT 3000 plus automated chemistry analyzer). GFR was also calculated using the CKD EPI equation. Full blood count was conducted on the sample in the EDTA tube with particular attention paid to the Hemoglobin (Hb) and Haematocrit (Hct) using Sysmex xs-500i automated hematology analyzer.

### Statistical analysis

Data collected was entered using Microsoft Excel using designed excel forms to avoid entry errors and analyzed with Statistical Package for Social Sciences (SPSS vs. 22.0). Descriptive statistics were used to calculate frequencies and proportions of study participants. Paired *t*-test was used to compare the means of biochemical variables. *p*-values less than 0.05 were considered statistically significant.

## Results

This study recruited 51 peasant farmers and hawkers whose modal age ranged between 25 and 29 years representing 52.94%. The lowest age of the participants was 22 and the highest was 35. Among them, 30 (58.82%) were males and 21 representing 41.18% were females (Table 1).

**Table 1:** Socio-demographic Features of Study Participants

Variable	Frequency	Percentage (%)
**Total**	**51**	**100**
**Age**		
20-24 yrs	6	11.76
25-29 yrs	27	52.94
30-34 yrs	11	21.57
35-39 yrs	7	13.73
**Sex**		
Male	30	58.82
Female	21	41.18

In this study, participant’s BMI and GFR were significantly higher during the wet season than in the dry season. Participant’s systolic blood pressure, creatinine, urea and potassium levels were significantly higher during the dry season than observed in the rainy season. Total body water (TBW), hemoglobin concentration, hematocrit and sodium levels were statistically similar among the participants during the two seasons (Table 2).

**Table 2:** Mean Seasonal Variations of Various Parameters among Study Participants

Variables	Rainy Season (Mean ± SD)	Dry Season (Mean ± SD)	***P***-Value
Body Mass Index (kgm^−2^)	25.14 ± 2.56	24.59 ± 2.69	**< 0.0001**
Systolic blood pressure (mmHg)	120 ± 8.60	121.88 ± 7.90	**0.0194**
Total Body Water	42.65 ± 4.86	41.86 ± 4.73	0.4097
Hemoglobin (g/dL)	13.49 ± 1.68	13.52 ± 1.68	0.7161
Hematocrit	41.14 ± 5.10	41.14 ± 5.10	0.7611
Creatinine (umol/L)	69.78 ± 19.99	90.51 ± 19.20	**< 0.0001**
Urea (mmol/L)	3.56 ± 1.22	4.00 ± 1.08	**0.0126**
Sodium (mmol/L)	138.98 ± 2.17	139 ± 1.85	0.1193
Potassium (mmol/L)	3.79 ± 0.25	3.85 ± 0.23	**0.0479**
GFR (ml/min/1.73m^2^ BW)	132.94 ± 21.40	105.67 ± 18.48	**< 0.0001**

*GFR* Glomerular Filtration Rate, *BW* Body Weight; *P*-value less than 0.05 is considered statistically significant

Findings revealed a significantly higher BMI among both males and females during the season of rains as against the dry season. Similarly, GFR among both males and females during the rainy season was significantly high per findings of this study. Conversely, significantly elevated creatinine levels among both males and females during the dry season were observed in this study. Differences in blood pressure and urea levels among males were significantly higher during the dry season than in the rainy season but however statistically comparable among the females. Potassium levels among both males and females during the two seasons were statistically similar per findings from this study (Table 3).

**Table 3:** Mean seasonal measurement of various study parameters stratified by gender

Variables	Rainy Season (Mean ± SD)	Dry Season (Mean ± SD)	***P***-Value
Body Mass Index			
Male	24.69 ± 2.13	23.99 ± 2.21	**< 0.0001**
Female	25.79 ± 2.99	25.45 ± 3.11	**0.0302**
Systolic blood pressure			
Male	121.27 ± 7.89	123 ± 7.02	**0.0205**
Female	118.19 ± 9.43	119.19 ± 8.46	0.4187
Creatinine			
Male	80.13 ± 17.18	103.57 ± 11.66	**< 0.0001**
Female	55 ± 13.44	71 ± 10.18	**< 0.0001**
Urea			
Male	4.05 ± 1.05	4.56 ± 0.97	**0.0302**
Female	2.87 ± 1.12	3.22 ± 0.66	0.2121
Potassium			
Male	3.87 ± 0.18	3.92 ± 0.18	0.1273
Female	3.68 ± 0.29	3.75 ± 0.27	0.2107
GFR			
Male	128 ± 22.13	98.40 ± 15.85	**< 0.0001**
Female	140 ± 18.59	116.05 ± 17.24	**< 0.0001**

*GFR* Glomerular Filtration Rate; *P*-value less than 0.05 is considered statistically significant

With the exception of participants within the age category of 35–39 years, significantly higher BMI was recorded among participants aged 20–24, 25–29 and 30–34 years during the rainy season than that observed during the dry season. Significantly elevated GFR was recorded among all age groups considered in this study in the rainy season as against the dry season. Participant’s creatinine levels compared to the rainy season was significantly higher among all age groups during the dry season. Systolic blood pressure as well urea levels were significantly higher among individuals aged 25–29 years during the dry season than were during the rainy season. Individuals aged 35–39 years also demonstrated significantly elevated urea concentration as during the dry season than were in the wet season. Potassium levels were statistically comparable among all the age categories during both seasons (Table 4).

**Table 4:** Age-Based Seasonal Variation among the Study Participants

Variables	Rainy Season (Mean ± SD)	Dry Season (Mean ± SD)	***P***-Value
**Body Mass Index**			
20-24 yrs	23.23 ± 3.20	22.38 ± 3.03	**0.0134**
25-29 yrs	24.75 ± 1.91	24.20 ± 1.98	**0.0002**
30-34 yrs	26.24 ± 2.92	25.69 ± 3.04	**0.0029**
35-39 yrs	26.57 ± 2.60	26.27 ± .3.03	0.2973
**Systolic blood pressure**			
20-24 yrs	117.5 ± 11.59	123 ± 11.24	0.1788
25-29 yrs	121.67 ± 6.65	123.56 ± 5.66	**0.0361**
30-34 yrs	119 ± 12.11	119.64 ± 11.56	0.7627
35-39 yrs	117.29 ± 6.24	118.00 ± 3.55	0.6556
**Creatinine**			
20-24 yrs	72.5 ± 32.47	105.17 ± 15.48	**0.0185**
25-29 yrs	78.56 ± 14.75	99.30 ± 15.28	**< 0.0001**
30-34 yrs	56.91 ± 17.68	72.09 ± 11.64	**0.0045**
35-39 yrs	53.86 ± 5.76	73.00 ± 9.71	**< 0.0001**
**Urea**			
20-24 yrs	5.33 ± 1.07	4.83 ± 0.98	0.3404
25-29 yrs	3.48 ± 1.04	4.30 ± 1.08	**0.0005**
30-34 yrs	3.31 ± 1.15	3.16 ± 0.74	0.7623
35-39 yrs	2.76 ± 0.74	3.48 ± 0.50	**0.0021**
**Potassium**			
20-24 yrs	3.95 ± 0.15	4.05 ± 0.08	0.1106
25-29 yrs	3.79 ± 0.26	3.85 ± 0.21	0.1578
30-34 yrs	3.71 ± 0.27	3.80 ± 0.27	0.1669
35-39 yrs	3.76 ± 0.24	3.77 ± 0.29	0.9258
**GFR**			
20-24 yrs	148.17 ± 8.04	119.17 ± 13.91	**0.0013**
25-29 yrs	133 ± 21.33	108.04 ± 18.81	**< 0.0001**
30-34 yrs	119.46 ± 19.60	94.91 ± 15.98	**0.0043**
35-39 yrs	140.86 ± 22.63	101.86 ± 17.02	**0.0034**

*GFR* Glomerular Filtration Rate; *P*-value less than 0.05 is considered statistically significant

**Fig. 1:**
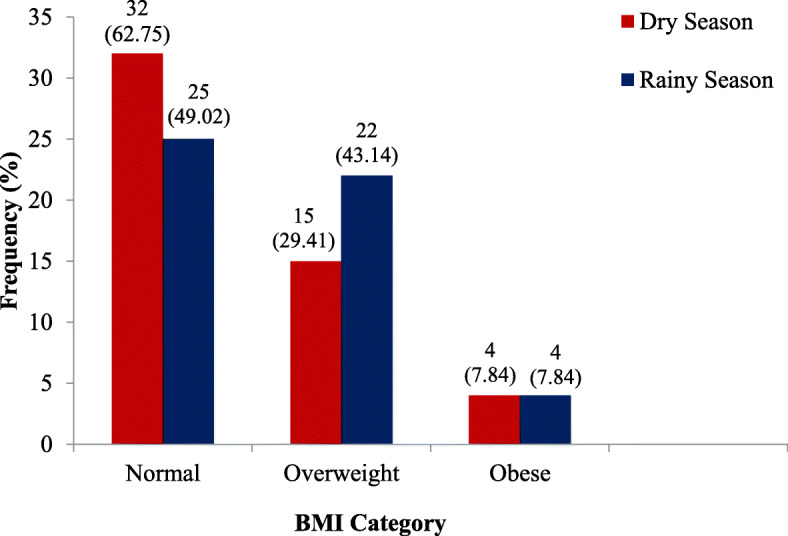
Participant’s BMI Stratified by the Two Seasons

Figure 1 shows BMI of participants by the two seasons. The BMI was categorized into normal (18.5–24.9), overweight (25.0–29.9) and obese (≥30). The number of participants with normal BMI rose from 49.02% in the rainy season to 62.75% during the dry reason. The contrary which saw more overweight (43.14%) participants in the rainy season as compared to the dry season (29.41%) was observed. The obese category for the two seasons was fairly the same.

**Fig. 2:**
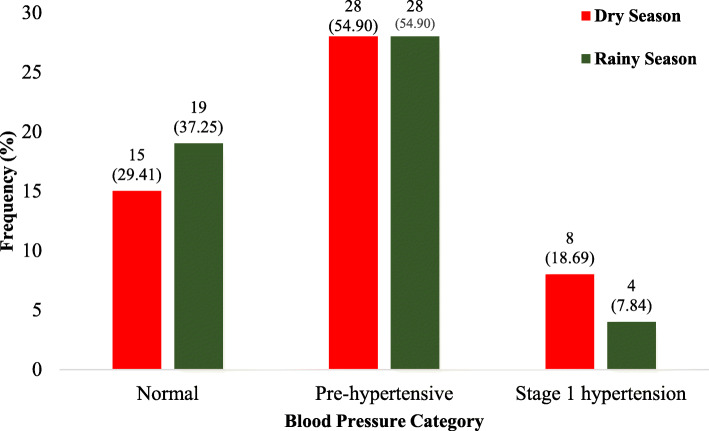
Participant’s Blood Pressure Distribution Stratified by the Two Seasons

Figure 2 shows the blood pressure distribution for the study participants over the two major seasons (rainy and dry season). In this study, normal blood pressure status was observed among 37.25% (19/51) of the participants in the rainy season more than 29.41% (15/51) of the participants in the dry season. About 55 % (54.90%) of the participants were pre-hypertensive both in the rainy and dry seasons. Meanwhile, stage 1 hypertension was preponderant in the dry season (15.69%) than that observed in the rainy season (7.84%).

## Discussion

To the best of our knowledge, this study is first of its kind to be done in Ghana and especially in one of the hottest regions of the country, Wa municipality. The study investigated the impact of climate variations on the kidney function of relatively young and healthy individuals in Wa, Upper West region of Ghana. The study ruled out as much as possible, traditional risk factors including hypertension, diabetes mellitus and therapy with cytotoxic agents including alcohol consumption [11–13] that could precipitate kidney disease among individuals. More males than females were recruited in this study. The preponderance of the participants (52.94%) were aged 25–29 years. This study revealed significantly elevated levels of serum creatinine (*p* < 0.0001), urea (*p* < 0.0126) and potassium (*p* < 0.0479) concentrations during the dry season compared to the rainy (wet) season. Additionally, significantly higher BMI (*p* < 0.0001), GFR (*p* < 0.0001) and systolic blood pressure (*p* < 0.0194) among the study participants during the rainy season than recorded during the dry season was also observed in this study. In the absence of kidney disease, consumption of high electrolyte containing foods and underlying causes of CKD, the discrepancies observed in the urea, creatinine and potassium concentrations despite the fact that these levels are within the normal ranges for a healthy individual may potentially be attributed to the excessive dehydration among the study participants. This is particularly true due to the nature of the dry season in the Wa Municipality which is trademarked by intense long, windy and hot dry periods. As a result, significantly reduced GFR among the participants during the dry season was paramount, further explaining the elevated levels of electrolytes observed in this study. Similar findings was observed among Marwari goats where mean serum creatinine was significantly (*p* ≤ 0.05) higher during hot period than the cold period [14] as well as among Mesoamerican sugarcane workers who demonstrated mean increase in serum creatinine of 0.21 mg/dl in Brazil, 0.12 mg/dl in El Salvador and 0.12 mg/dl in Nicaragua after a heavy workday with intense hot weather in focus [15]. This study recorded no significant seasonal alteration in the hematocrit and hemoglobin parameters of the study participants. There is absolute absence of scientific data to explain this observation among humans especially when a contradictory outcome has been reported among broilers. In a study conducted as far back as 1971 to assess the effect of environmental temperature on hematocrit and hemoglobin values of chicks receiving diets containing various levels of copper (Cu) and iron (Fe), it was revealed that an increase in temperature caused a corresponding decrease in the hemoglobin and hematocrit levels of broilers [16]. Well, perhaps the cupper and iron components of the study played a role in the variation of the parameters studied. A much in-depth study involving a larger sample size will perhaps reveal the true effect of temperature on these two parameters among humans. In this study, most of the parameters assessed were largely altered among the males than the female group. Except for potassium levels, significant drop in BMI and GFR as well as elevated creatinine and urea levels coupled with high systolic blood pressure among males in the dry season was dominant. This phenomenon could be due to the fact that men of Wa and the Northern Region in general are usually exposed to the hot sun during their business activities such as mobile sales of second hand clothing, beads, scrap dealing and the dominating motor cycle business popularly known as the okada business. Other businesses such as rearing of cattle and other animals which requires that the owners take the animals to the field for grazing or bring to the animals feed from the field can also be pinpointed as a cause of the discrepancies observed on the parameters among the males as their frequent exposure to the high temperature renders them dehydrated for longer periods. These activities expose the men to high temperatures which has been established to frequently lead to water scarcity in tropical regions raising the risk of dehydration suspected to have a direct link to CKD [9, 13, 17]. It is also important to note that, some of the women in the Wa municipality do engage in sales of women clothing and related items (hawkers) and rearing of farm animals requiring them to just like their male counterparts be mobile during their business hours hence exposing them to hash harsh weather conditions. This could explain the significantly elevated creatinine and reduced GFR observed among the females in the dry season. It is unclear whether the elevated serum creatinine observed among the participants during the dry season could also be attributed to the high consumption of meat among the study participants throughout the seasons. Meanwhile, it can however be argued that high muscular activities characterized by the peculiar business types engaged in by the study participants in synergy with dehydration could account for the increased serum creatinine observed during the dry season. Reduced GFR as well as elevated serum creatinine levels across all age groups studied was observed during the dry season. In addition, systolic blood pressure and urea levels were significantly higher among individuals aged 25–29 years during the dry season than were during the rainy season. The characteristic dehydrative effect and lack of frequent consumption of fluid during these periods of dryness with possible heat waves explains these findings. Meanwhile the age differences could merely be due to the fact that majority of the participants recruited for this study fell within the age bracket of 25–29 years, most of which were males who exposed themselves more to the hot weather than their female counterparts as enumerated above. Though not evaluated, owing to the variation in CKD biomarkers assessed in this study, possible initiation of kidney disease such as acute kidney injury (AKI) due to severe dehydration may also account for the observed reduced GFR and elevated serum creatinine levels across all age groups. Findings from this study indicates largely a normal BMI among the participants in both seasons. Overweight status of 43.14% was observed among the study participants during the raining season than 29.41% in the dry season. Authors could not put a finger on a concrete reason behind this observation. However, authors postulate that though not assessed in this study, increase in body fat (contributing to overweight and obesity if not controlled) during the wet season as a consequence of a possible reduced vitamin D levels [18, 19] from lack of exposure to adequate amount of sunlight may have played a role in the observations made. Prevalence of obesity, 4 (7.84%) was identical among the study participants in both seasons. This could be due to the fact that not all the participants observed in this study were highly active in terms of their business activities. A select few lived sedentary lives such as sitting at one place throughout the day while conducting their businesses and not exposed directly to the high temperatures compared to the others whose business life entailed moving from one locality to the other, exposing themselves to varied climate conditions. Such sedentary life habits by this group of individuals in combination with poor dietary behaviors such as consumption of more caloric food could account for their obese status in both seasons. In fact, the people of Wa and the Northern Region of Ghana in general are known to be people whose meal seldom lack either of those animal products such as meat from cattle, sheep, goat, guinea pig among others.

The season-wise stratification of participant’s blood pressure analyzed in this study revealed a pre-hypertensive status among 54.90% of the total participants studied in both seasons. Stage 1 hypertension was diagnosed among 15.69% of the total participants in the dry season more than 7.84% diagnosed among same participants in the wet season. This finding seems to hold a hidden trend of disease progression as pre-hypertension was observed among preponderance of the participants in both seasons and yet stage 1 hypertension was high during the dry season than the wet season. This could mean that, frequent exposure to adverse climatic conditions, vis-à-vis hot temperatures could be a gradual fueling stimulus for the increase in prevalence of hypertension among the people of Wa municipality in the foreseeable future.

## Conclusion

This study suggests that exposure to hot climatic weather conditions could lead to kidney diseases as significant variations in biomarkers of kidney impairment were observed. Worth nothing is that both genders are affected equally when exposed to the warm climatic conditions. High BMI was however not a prerequisite factor to developing kidney diseases associated with hot climatic conditions. The data also suggest that hypertension may occur from exposure to higher temperatures during the dry season. Public health education on the dangers of the over exposure to the various climatic conditions and the need to frequently rehydrate avert the hostility of the harsh climate when exposure becomes unavoidable.

## Limitations

Factors such as rhabdomyolysis and inflammation that could cause increased serum Creatinine and urea levels were not assessed. Limited sample size was employed in this study which could account for some of the insignificant variations observed in some of the parameters in this study. This study employed non-standardized method of creatinine estimation and that could affect the general outcome of the results obtained in this study. It must also be noted that, this study did not account for the effects of regression to the mean (RTM); as a result, the variation observed could be due to the outcome of RTM. Additionally, only single measurements for anthropometric and biochemical parameters were obtained in each season. Finally, the study hypothesises that dehydration in the dry season may be implicated, but this is yet to be proven. This therefore calls for further studies on the topic.

## Data Availability

The data are available from the corresponding author on reasonable request.
